# Compact Urban Form and Human Development: Retest Based on Heterogeneous Effects

**DOI:** 10.3390/ijerph19042198

**Published:** 2022-02-15

**Authors:** Lu Liu, Yu Tian

**Affiliations:** School of Business, Sun Yat-sen University, Guangzhou 510275, China; liulu68@mail2.sysu.edu.cn

**Keywords:** compact city, human development, urban shape

## Abstract

The Human Development Index does not follow a normal distribution. For skewed distributions, finite mixture models can provide better estimates than fixed-effects models. In this paper, the relationship between compact cities and human development is investigated by employing a finite mixture model using panel data of Chinese prefecture-level cities. In contrast to the majority of the literature, which focuses exclusively on economic density, this article examines the impact of economic and morphological density on the level of human development. The results show that the compact development model has a negative impact on the level of human development and that the intensity of the impact varies for cities with different characteristics.

## 1. Introduction

Human development (defined as enlarging people’s choices in a way that enables them to lead longer, healthier, and fuller lives) has gradually replaced economic growth as the ultimate goal of human activities since the twenty-first century. Where a choice is required, human development should be prioritized in terms of sequencing [1]. Scholars and policymakers are concentrating their efforts on discussing the various factors that influence human development in order to accomplish this goal [1,2,3,4,5,6,7,8]. Among the discussions, there is a particularly heated debate about the impact of compact cities on sustainable human development.

Proponents of the compact city concept promote high-density (e.g., economic density, morphological density) and mixed-use developments (e.g., co-location of residential, commercial and retail uses) as the critical solutions to countervail the negative externalities of urban sprawl and to improve human development. Relevant empirical evidence indicates that compact urban form has a positive effect on a variety of outcomes, including improved accessibility, improved economic outcomes (e.g., productivity, innovation), reduced energy consumption, increased efficiency of public service delivery, and improved health, safety, subjective well-being, and social equity [9,10,11,12,13,14,15,16]. According to this evidence, we can infer that compact cities may promote human development. However, not everyone appreciates the compact city model. Opponents pointed out the negative aspects of compact cities, such as shortages in urban green spaces, failures in providing affordable housing, and overcrowding in the urban residential area [9,11,12,15,17,18,19]. These negative aspects, in turn, suggest that compact cities may have a negative impact on human development. Therefore, it is unclear if the compact city model will promote human development.

In addition, some scholars have suggested that the polarizing debate over compact cities may be the result of over-focusing on the experience of high-income countries, in particular, those in North America, while ignoring other urban backgrounds [20,21]. Unlike cities in high-income countries with low density and mature urbanization stages, many cities are representatives of high density and are experiencing rapid urbanization. Numerous high-density cities can be found in East and Southeast Asia. These cities have decided to develop in a high-density way emphasizing the benefits of the compact urban form. However, the benefits or trade-offs of this urban form on human development are yet to be validated. It is time to review the validity of such a development model in view of the considerable increase in urban compactness in many East and Southeast Asian cities over the last few decades.

In this research, by selecting an exemplary, high-density country, we would have a chance to validate the claims on the benefits of compact form on human development. Using panel data for 285 cities in China from 2000 to 2018, we calculated the compactness of city shape and constructed a human development index (hereafter HDI) for each city to model the relationship between compactness and human development. By decomposing the HDI, we explore possible influence channels through which compactness affects human development, providing additional empirical evidence for the ‘compact city’ debate. In contrast to previous arguments that compact cities can contribute to human development, the main conclusion of this study is that compact development in a high-density context can have a negative impact on human development. Both increased economic density and increased morphological density pose serious challenges to human development.

The main contributions of our study can be summarized in three aspects. (1) The values of HDI does not follow a normal distribution; estimating only the average effect of the full sample may lead to biased estimation results. Therefore, we use mixtures of distributions to model the relationship between compactness and human development to refine the estimates. By employing this methodology, we could identify the heterogeneous effects. (2) Previous research has primarily focused on North American cities, ignoring the role of compactness in other context. In this paper, we use panel data from Chinese cities to examine the effect of compactness on human development. By utilizing this data, we could provide additional empirical evidence on the socioeconomic impacts of compactness in high-density cities. (3) A lot of empirical studies examine the outcomes of economic density but ignore the outcomes of morphological density. In contrast to previous research, this study employs night light data to quantify morphological density (i.e., the compactness of urban footprints), thereby compensating for a dearth of empirical research on morphological density.

The remainder of our paper is organized as follows. Section 2 introduces the model, methodology, and data. Section 3 reports the empirical results, covering the heterogeneous effects of compactness on human development and some robustness tests. Section 4 discusses the significant findings and concludes. Section 5 describes the limitations of the study.

## 2. Econometric Specifications and Data

### 2.1. Traditional Model Specification

We begin by examining the total effect of city compactness on human development using a traditional panel data method, namely the fixed effects model. Consistent with previous research on the determinants of human development [3,22], this paper empirically examines the effect of compact city form on human development by estimating the following equations.

(1) $$hdi_{i,\;t}=\alpha *nCohesion_{i,t}+\gamma_{1}*C_{i,t}+\delta_{i}+\epsilon_{t}+\varepsilon_{i,t}$$

where $hdi_{i,\;t}$ is the human development index, $nCohesion_{i,t}$ is the normalized cohesion index, and $\delta_{i}$, $\epsilon_{t}$ are city fixed effect and year fixed effect. $C_{i,t}$ consists of a series of control variables, including the proportion of fiscal expenditure (*fiscal*, the ratio of fiscal expenditure to gross domestic product (hereafter GDP)), trade openness (*ftd*, the ratio of the sum of the exports and imports of the cities to GDP), house prices (*lnHsPr*, the ratio of sales to sales area of commercial housing, deflated by Consumer Price Index (CPI) and in log), industrial structure (*IndStr*, the ratio of tertiary sector added value to secondary sector added value), and economic density (*density*, number of resident population per unit area). Note that *i* (*i* = 1 … 285 cities) and *t* (*t* = 2000 … 2018 years) denote city and year, respectively.

### 2.2. Finite Mixture Model

A finite mixture model (hereafter FMM) is a statistical model that presupposes the existence of unobserved groups within a population, referred to as latent classes. Each latent class has its own regression model that can be fitted. As a result, FMMs are widely used to model and analyze heterogeneous data sets with skewness [23,24].

To test the heterogeneous effects of city compactness on HDI at the prefecture-level, the finite mixture model (see Equation (2)) with covariates was established in accordance with the basic model mentioned above. We include two variables that may be useful for grouping: geographical location (*region*) and city size (*level*). As for geographical location, the cities are classified into four areas based on their location: western, central, northeast, and eastern. The cities in the western, central, northeast, and eastern areas are denoted by 1, 2, 3, and 4, accordingly. The larger the value, the more advantageous the geographical location. As a result, cities with smaller values are farther away from the coastlines. In terms of city size, cities are classified into six categories based on their commercial attractiveness. The detailed categories can be found at https://www.yicai.com/news/101063860.html (accessed on 5 December 2021). The six categories categorised in ascending order of commercial attractiveness are denoted as 5, 4, 3, 2, 1 and 0, respectively.

(2) $$f\left(hdi|nCohesion,\;cv,\Theta \right)=\overset{G}{\underset{g=1}{\sum }}\pi_{g}\left(cv,\;\alpha_{g}\right)f_{g}(hdi|nCohesion;\;\beta_{g},\sigma_{g})$$

where Θ is the unknown parameter vector, G is the number of groups, $\beta_{g}$ are the unestimated coefficients of explanatory variables in group g, cv are the covariates, and $\alpha_{g}$ are the corresponding parameters to be estimated. $f_{g}(hdi|nCohesion;\;\beta_{g},\sigma_{g})$ represents the conditional distribution of explained variables in group g. Specifically, $\sigma_{g}$ is the standard deviation of error in group g, and $\pi_{g}$ is the probability of a city belonging to a particular group g, which follows the following multinomial logit model [25]:

(3) $$\pi_{g^{\prime }}\left(cv,\;\alpha_{g^{\prime }}\right)=\frac{exp\left(\alpha_{g^{\prime }}+cv*\alpha_{g^{\prime }}\right)}{\sum_{g=1}^{G}exp\left(\alpha_{g}+cv*\alpha_{g}\right)}$$

The maximum likelihood estimation method is used to estimate the coefficients in Equation (2), and the likelihood function of Equation (2) can be written in a logarithmic form as follows:

(4) $$logL=\sum_{n=1}^{N}log\left[\sum_{g=1}^{G}\pi_{g}\left(cv,\;\alpha_{g}\right)\prod_{t=1}^{T}f_{g}\left(hdi_{i,t}|\;nCohesion_{i,t},\;\beta_{g},\sigma_{g}\right)\right]$$

The parameter estimator of the finite mixture model can be obtained by estimating the above equation using the Expectation–Maximization algorithm. Further, by using the Empirical Bayes method, the posterior probability of city *i* belonging to a specific group *g*’ is calculated as follows:

(5) $${\hat{\pi }}_{ig^{\prime }}=\frac{\pi_{g^{\prime }}\left(cv_{i},\hat{\alpha_{g^{\prime }})}f_{g^{\prime }}(y_{i}|X_{i};\;\hat{\beta_{g^{\prime }}},\hat{\sigma_{g^{\prime }}}\right)\;}{\sum_{g=1}^{G}\pi_{g}\left(cv_{i},\hat{\alpha_{g})}f_{g}(y_{i}|X_{i};\;\hat{\beta_{g}},\hat{\sigma_{g}}\right)}\;$$

All cities can be grouped based on the probability calculated by the above equation. At this point, the overall probability and overall classification error for all cities belonging to group *g*′ are given by the following equations, respectively.

(6) $$P_{g^{\prime }}=\frac{\sum_{n=1}^{N}\hat{\pi }(g^{\prime }|cv_{i},y_{i})}{N}$$

(7) $$error=1-\frac{\sum_{n=1}^{N}max\hat{\pi }(g^{\prime }|cv_{i},y_{i})}{N}$$

It should be noted that before estimating FMM, the optimal grouping number *G* must be determined. The Akaike Information Criterion (hereafter AIC) and Bayesian Information Criterion (hereafter BIC) are used in this paper to accomplish this. The following is the corresponding calculation equation:

*BIC* = −2*LL* + *Jlog*(*N*) (8)

*AIC* = −2*LL* + 2*J* (9)

In Equations (8) and (9), *LL* is the log-likelihood value calculated by (4), *J* represents the parameter matrix, and *N* represents the total number of observations.

### 2.3. Variables, Data Sources, and Summary Statistics

The variables are described in detail below. Two datasets were used to investigate the effect of city compactness on human development: the Night-time Lights dataset and the HDI dataset.

First, we used the DMSP/OLS Night-time Lights dataset (recorded using an Operational Linescan System (OLS) from the U.S. Air Force Defense Meteorological Satellite Program (DMSP)) for 2000–2013 and the NPP/VIIRS Night-time Lights dataset (recorded using the Visible Infrared Imaging Radiometer Suite (VIIRS) on the Suomi National Polar-orbiting Partnership (NPP) satellite) for 2014–2018. The dataset contains satellite images of the earth at night that record the intensity of Earth-based lights each year. We delineated the urban footprints using the images by considering spatially contiguous lighted pixels surrounding a city’s coordinates that have a luminosity greater than a predefined threshold of 10. We then quantified the shape compactness of the urban footprints for each year. Shape compactness is defined as the degree to which a polygon’s shape deviates from a circle. Numerous indexes can be used to measure the shape compactness; for this study, we used the cohesion index, an indicator used in urban planning [26,27].

Cohesion is a natural measure of the overall accessibility of metropolitan areas. The cohesion property of geographic shapes focuses on the proximity of objects to one another. The more cohesive the metropolitan area, the better the accessibility it offers its inhabitants. The cohesion index is defined using the average Euclidean distance (in kilometers) between two points within a polygon. Higher values of the cohesion index mean longer distances between points and less compact shapes. Note that any compactness index based on distance within a polygon is mechanically correlated with the polygon area. The footprint area was controlled in the computation of the cohesion index to disentangle the geometry effect from the city size. In addition, it is important to highlight that the normalized cohesion index, *nCohesion*, is used throughout this paper.

We then used the HDI dataset. The human development index is given by the expression:

(10) $$hdi=\sqrt[{3}]{stv_{hb}*\;stv_{ays}*stv_{gdp}}$$

where $stv_{hb}$ represents the standardized value (The min–max standardization approach is used to make variables measured at different scales comparable) of the number of hospital beds per capita, $stv_{ays}$ represents the standardized value of average years of schooling, and $stv_{gdp}$ represents the standardized value of GDP per capita.

**Figure 1:**
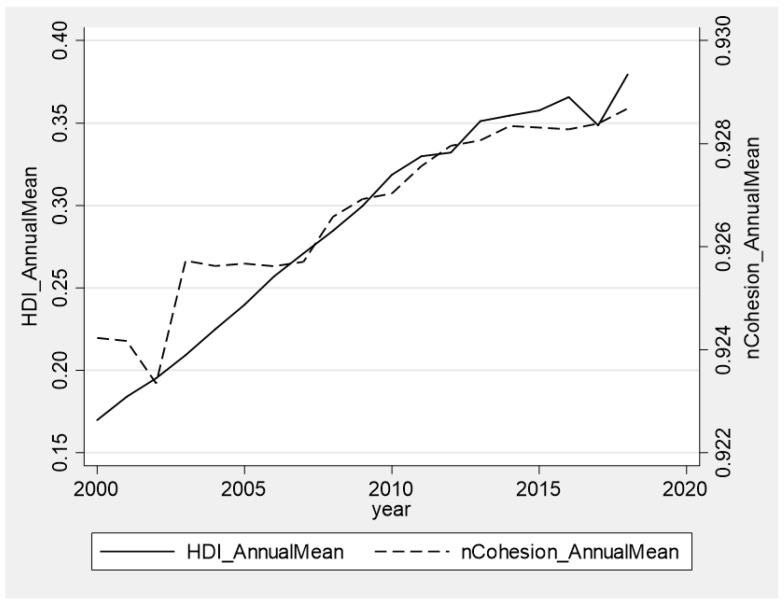
Trends in shape compactness and HDI from 2000 to 2018. Notes: The left vertical axis is the annual mean of HDI, and the right is the annual mean of *nCohesion*. On the horizontal axis is the year.

To clarify the long-term trend of the city compactness and human development index, we created a time-series graph (see Figure 1). In general, both the city compactness and human development indices increase over time.

To account for the effects of extraneous variables, we included several control variables: *fiscal*, *ftd*, *IndStr*, *lnHsPr*, and *density*. These data were obtained from China City Statistical Yearbook, China Statistical Yearbook For Regional Economy, and statistical yearbooks at the provincial and city levels. Due to some missing values, we also referred to other data sources, such as the Wind Economic Database and the CEIC Data (https://insights.ceicdata.com/node/CN, accessed on 5 December 2021). Additionally, we introduce the relief degree of land surface (hereafter rdls) in the robustness test. The relief degree of land surface is an important factor in the macroscopic description of landforms and is often used as a control variable in economic studies. The data were obtained from http://www.geodoi.ac.cn/WebCn/doi.aspx?Id=887 (accessed on 5 December 2021). The descriptive statistics of variables are shown in Table 1. The correlation coefficient matrix of the variables is shown in Table 2. As can be seen from Table 2, there is no serious problem with multicollinearity.

## 3. Empirical Results

In Section 3, we estimate the parameters of the aforementioned regression models using the fixed effects model (hereafter FE) and the finite mixture model, with a focus on the estimation results of the finite mixture model.

### 3.1. Implications of Compactness for Human Development

Table 3 present the estimated results of the fixed effects model reflecting the average impact of urban compactness on human development. The results show that the coefficient for *nCohesion* in Column (1) is not significant. Here, we cannot simply assume that urban compactness has no impact on human development. It is possible that the insignificance of the coefficients is due to poor model setting. According to Doğru (2017) [24], HDI does not necessarily obey a normal distribution. The results of fixed effects can be biased when the dependent variable does not obey a normal distribution. Therefore, we tested the normality of the HDI. The result of the Shapiro–Wilk normality test shows that the *p*-value is 0.00000, rejecting the null hypothesis of a normal distribution. It confirms that HDI does not follow a normal distribution and that the total sample could be a mix of a finite number of normal distributions. Under this circumstance, a better estimate could be obtained with FMM. It is necessary to estimate the finite mixture model.

The number of latent groups is unknown prior to estimating the finite mixture model. We use the information criterion to select the best-fitting model, and the preferred model is the one with the minimum information criterion value. Table 4 show the results of the finite mixture model’s group testing. As can be seen from the table, the higher the number of groups, the smaller the criterion value. Therefore, we should choose the model with as many groups as possible. However, the coefficient difference between groups is not significant when separated into four/three groups hence the finite mixture model divided into two groups is eventually chosen for estimates.

Table 5 and Table 6, respectively report the estimation results and descriptive statistics in each group. The main findings are as follows:

Cities in different groups have diverse urban features, as seen in Table 6. We have given each group a name based on these qualities for ease of expression. Because the cities in the first group have lower per capita income, lower house prices, lower population density, and less foreign trade, we label them the backward city group. The second group is labeled as the advanced city group, and it contains characteristics that are opposed to Group 1, such as high per capita income, high population density, high house prices, established industries, and so on. The following are the estimated results for these two groups.

Firstly, the coefficient of economic density reflected by population density on human development is negative for cities with various features, which contradicts prior study findings. Although the spatial concentration of economic activity in urban areas is relatively high, some experts argue that a higher density of urban growth is beneficial on average [28,29]. However, the results of this study show that excessive pursuit of urban economic density is detrimental to the advancement of human development level, according to the empirical examination of high-density cities.

Secondly, for cities with different characteristics, the coefficients of *nCohesion* on human development are also negative. The symbols of coefficients are the same for the two groups. However, by comparing the coefficient sizes between the two groups, it is found that the coefficients of the two groups are significantly different. We conclude that among cities with developed economies (higher trade openness and senior industry), urban compactness has a greater negative impact on the level of human development.

Given that the distribution of economic activity may also be affected by human development, we use the instrumental variable methods to re-estimate the finite mixture model. The compactness of a city’s administrative area is used as an instrument for *nCohesion*, as it is highly predictive of the spatial distribution of economic activity in cities but uncorrelated with HDI. Like the aforementioned shape compactness of economic activity, the normalized cohesion index of the administrative division (*Adm_nCohesion*) reflects the extent to which the contour of administrative division deviates from the circle. Table 7 show the re-estimation results. Both the coefficients of *nCohesion* and *density* are consistent with the original estimation result, and the fundamental results are robust. Economic and morphological density has a detrimental effect on human development.

The relief degree of land surface may affect the shape of the distribution of urban economic activities and thus the level of human development. In order to eliminate the influence of terrain on HDI, this paper introduces the relief degree of land surface as control variables into the benchmark model. The re-estimation results are given in Table 8. The estimated result is robust.

### 3.2. Implications of Compactness for Different Dimensions

We then analyzed the relationship between city compactness and the three dimensions of the HDI (education, healthy, and standard of living). The purpose of this section was to explore whether the effect of city compactness on different dimensions of human development will change over different city environments.

Table 9 show the influence of city compactness on the educational dimension. For both groups, the increase in shape compactness has a significant negative impact on education. Besides, cities in Group 2 are more strongly affected than Group 1. Combining the comparison of the characteristics of the two groups, it can be found that the more advanced and high-density the cities are, the larger the negative effect of compact development on education. This could be a result of the mismatch in educational resource supply and demand generated by compact development. Scholars generally agree that families are closer to facilities in a compact city [11,12], but it is worth noting that compact city shape is associated with faster population growth [27]. The population is disproportionately concentrated in specific areas, but educational opportunities are often limited, making it difficult for people to access educational resources in compact cities. Similar to morphological density, the effect of population density on average years of schooling is also negative, and this negative effect is also larger for Group 2 than Group 1.

Table 10 show the effect of compact cities on the health dimensions. The coefficients of *nCohesion* are significantly negative in both column 1 and column 2. Likewise, the coefficients of *density* are both significantly negative. This implies that the increase in both morphological density and economic density has a negative impact on the health dimension. Increased density leads to increased competition in health care resources, and there may be a mismatch between population distribution and available health care resources. Proponents of compact cities claim that compact cities are good for the health of their residents. On the one hand, compact cities increase opportunities for walking and bicycling by reducing average travel distances [11,12], which is beneficial to the health of residents. On the other hand, compact cities reduce car use and thus reduce CO_2_ emissions [16]. As air pollution decreases, respiratory diseases such as asthma and lung cancer are greatly reduced, which also has a beneficial effect on the health of the population. What proponents of compact cities overlook, however, is the impact that urban form may have on the health of the population through the accessibility of healthcare resources. Although compact urban development may have a positive impact on residents’ health through reduced automobile use, it is also important to note that compact cities are prone to a shortage of medical resources, which can negatively affect residents’ health.

Table 11 show the influence of city compactness on the standard of living. The explained variable is the GDP per capita. In terms of the effect of morphological density, the coefficient of *nCohesion* for Group 2 is positive, while the coefficient for Group 1 is negative. This suggests that not all cities have been able to achieve economic growth and higher incomes through compact forms. We conjecture that compact development has the potential to promote economic growth and living standards of residents in cities with advanced industrial structures and high trade openness, while the concept of compact cities is not feasible in underdeveloped cities. However, due to the insignificance of the regression coefficient in this article, the conclusion requires additional testing.

Another important finding is that the coefficient of *density* is significantly negative for both two groups. This means that the higher the economic density, the lower the standard of living. That is, in high-density Chinese cities, pursuing higher economic density is unfavorable. Furthermore, comparing the coefficients of *density* between the two groups reveals that the negative impact is stronger for Group 1 than for Group 2. Combining the different characteristics of the two groups, we find that the impact of economic density on the standard of living is related to the role of cities in macroeconomic growth. The negative effect of economic density on the standard of living is weaker for cities with advanced industrial structures and a high degree of trade openness. This finding is consistent with the prevailing view in urban economics that cities that approach the growth frontier could support higher population densities. It has been thought that the impact of population density on economic growth is generally non-linear [30,31,32,33,34,35] and that the inflection point is closely related to the institutions [36], urban governance capacity [37,38,39], infrastructure [40], and stage of economic development [41]. The results of this study, while confirming this prevailing view, also suggest that the inflection point may be related to density itself.

## 4. Discussion and Conclusion

Whether an advocate for compact development or for low-density development, the majority of the arguments are based on an examination of the expansion process of low-density cities. There is relatively little literature examining the impact of compactness on human development in the context of high-density cities. Besides, the majority of previous research has investigated the average effect of compact development, oblivious to the heterogeneous characteristics of urban areas. This paper, therefore, examines the impact of compactness on human development in the context of high-density cities, with due regard to the heterogeneous characteristics of different cities, to provide additional empirical evidence on this issue. The empirical results indicate that in the presence of sample heterogeneity, the average effect calculated by the fixed effects model is biased, masking the heterogeneous effects that exist in reality.

Firstly, some scholars pointed out that although the spatial concentration of economic activities in urban areas is already high, on average, a higher density of urban development is desirable. This view differs from the results of this article. The empirical analysis of this paper based on the background of high-density cities shows that the pursuit of urban compactness is not good for the improvement of human development. High morphological density does not help inhabitants improve their educational levels, exacerbates the shortage of medical resources, and decreases residents’ quality of life. Similarly, the increase in economic density is also detrimental to improving human development, with effects roughly similar to morphological density. This suggests that a frequent challenge for cities in the process of compact development is the mismatch between the distribution of public services and the population. To effectively reduce congestion induced by population concentration, the spatial distribution of public services (e.g., schools, hospitals, etc.) must be properly designed. This puts forward a high request for the level of integrated urban construction, and if a city fails to meet the request, it will be unable to truly advance the city’s human development, even if it achieves compact development.

Secondly, the effect size of compact cities on human development and its different dimensions varies with city characteristics. While advanced cities may be able to mitigate the negative economic effects of congestion in compact cities through agglomeration economies, they appear helpless when it comes to providing public services such as education and health care. The findings of this paper suggest that urban compactness should be considered in the context of the role of cities in economic growth, rather than simply looking at the experiences of other countries or examining the impact of urban compactness in an average sense, which may not be conducive to a deeper understanding of the role of compact patterns in promoting human development.

## 5. Research Deficiency and Prospect

Firstly, compact cities include both the pursuit of optimal density (economic density and morphological density) and the mixed use of land. However, this article, limited by the availability of data, does not consider the impact of mixed use of land on human development. In future research, taking into account the various characteristics of compact cities and effectively testing the interaction between multiple characteristics will help draw better scientific conclusions.

Secondly, economic phenomena are intrinsically specific and very sensitive to changes in the external environment. If the correct causal relationship cannot be found, the subsequent policy design may be ineffective, and the effect may even be counterproductive. By focusing on the heterogeneous characteristics of cities, this article analyzes the impact of compact cities in more detail. Monkkonen et al. (2020) [42], in their research on the economic results of compact cities, also focused on the heterogeneity of the productivity of manufacturing and service industries and reached a different conclusion from previous studies, pointing out that the compact urbanization policy agenda needs to consider the country’s economic structure and manufacturing needs. This inspires us to pay more attention to the issue of heterogeneity in the study of compact cities, which may provide more clues to explain the current research debates.

## Figures and Tables

**Table 1:** Descriptive statistics.

Variable	Observations	Mean	Std. Dev.	Min	Max
*hdi*	5364	0.177	0.0923	0	0.531
*pchb*, beds/10 thousand people	5364	35.28	15.01	5.770	180.7
*lnays*, years	5364	2.041	0.0666	1.870	2.340
*lnpcgdp*, one thousand CNY	5364	2.871	0.822	0.501	5.193
*nCohesion*	5364	0.927	0.0524	0.705	0.996
*IndStr*	5364	0.896	0.449	0.0943	4.894
*ftd*	5364	0.199	0.390	4.16 × 10^−8^	5.443
*fiscal*	5364	0.144	0.0962	0.00182	1.027
*lnHsPr*, in log	5364	7.714	0.600	5.499	10.53
*density*, 10,000 person per square kilometer	5364	0.0457	0.0499	0.000498	0.652
*Adm_nCohesion*	5364	0.866	0.0727	0.516	0.974
*rdls*	5364	0.666	0.745	0.00132	3.814
*level*	5364	3.609	1.229	0	5
*region*	5364	2.448	1.200	1	4

Notes: Std. Dev. represents standard deviation.

**Table 2:** Correlation coefficient matrix of variables.

	*hdi*	*pchb*	*ays*	*lnpcgdp*	*nCohesion*	*density*	*IndStr*
*hdi*	1						
*pchb*	0.7707 *	1					
*ays*	0.7887 *	0.5148 *	1				
*lnpcgdp*	0.8985 *	0.5989 *	0.5752 *	1			
*nCohesion*	0.0258 *	−0.0709 *	0.0003	0.0939 *	1		
*density*	0.2098*	−0.0546 *	0.1442*	0.3129 *	0.0960 *	1	
*IndStr*	0.0737 *	0.1003 *	0.1944 *	−0.0912 *	−0.0384 *	0.0408 *	1
*ftd*	0.2243 *	−0.0173	0.1425 *	0.3470 *	0.1264 *	0.5709 *	0.0549 *
*fiscal*	0.0715 *	0.2200 *	0.0269 *	0.0452 *	−0.0299 *	−0.1979 *	0.2584 *
*lnHsPr*	0.7539 *	0.4797 *	0.5348 *	0.8131 *	0.1132 *	0.4135 *	0.1631 *
*level*	−0.4246 *	−0.1255 *	−0.4782 *	−0.4136 *	−0.2060 *	−0.5680 *	−0.1318 *
*region*	0.1858 *	−0.0529 *	0.1119 *	0.3223 *	0.2005 *	0.3680 *	0.0444 *
	*ftd*	*fiscal*	*lnHsPr*	*level*	*region*		
*ftd*	1						
*fiscal*	−0.1286 *	1					
*lnHsPr*	0.3705 *	0.2280 *	1				
*level*	−0.4521 *	0.3112 *	−0.4919 *	1			
*region*	0.3736 *	−0.2932 *	0.3419 *	−0.4159 *	1		

Notes: * represents *p* < 0.1.

**Table 3:** Impact of city compactness on human development: fixed-effect model.

	(1)
	FE
*nCohesion*	0.0597
	(0.98)
*density*	−0.00477
	(−0.03)
*IndStr*	0.00134
	(0.33)
*ftd*	0.00302
	(0.63)
*fiscal*	−0.138 ***
	(−6.46)
*lnHsPr*	0.00405
	(1.20)
*_cons*	0.00813
	(0.14)
r2_a	0.8839
F	199.5900 ***
N	5364

Notes: t statistics are in parentheses. *** represents *p* < 0.01. For concise presentation, the coefficients for the year dummies are excluded from the tables.

**Table 4:** The information criterion value.

Model	Observations	Log Likelihood (Model)	DF	AIC	BIC
fmm1_hdi	5364	7677.917	8	−1.53 × 10^4^	−1.53 × 10^4^
fmm2_hdi	5364	8411.165	19	−1.68 × 10^4^	−1.67 × 10^4^
fmm3_hdi	5364	8600.761	30	−1.71 × 10^4^	−1.69 × 10^4^
fmm4_hdi	5364	8747.996	41	−1.74 × 10^4^	−1.71 × 10^4^

Note: AIC and BIC refer to Akaike information criterion and Bayesian information criterion, respectively. Observations are used in calculating BIC. DF refers to degrees of freedom.

**Table 5:** Impact of city compactness on human development: fmm.

	(1)	(2)
	1b. Class	2. Class
*level*	0	−0.889 ***
	(.)	(−14.39)
*region*	0	−0.489 ***
	(.)	(−8.70)
*_cons*	0	3.595 ***
	(.)	(12.08)
*nCohesion*	−0.0394 ***	−0.272 ***
	(−2.64)	(−8.79)
*density*	−0.276 ***	−0.528 ***
	(−11.77)	(−13.38)
*IndStr*	−0.0240 ***	−0.00508
	(−12.13)	(−1.51)
*ftd*	0.0125 ***	−0.0196 ***
	(3.24)	(−4.47)
*fiscal*	−0.0146	−0.307 ***
	(−1.61)	(−8.85)
*lnHsPr*	0.0913 ***	0.184 ***
	(50.08)	(51.55)
*_cons*	−0.480 ***	−0.883 ***
	(−25.94)	(−23.83)
*var(e.hdi)*	0.00143 ***	0.00306 ***
	(27.10)	(23.49)

Notes: z statistics are in parentheses. *** represents *p* < 0.01.

**Table 6:** Descriptive statistics in each group.

Variable	Group 1		Group 2	
	Mean	Std. Dev.	Mean	Std. Dev.
*hdi*	0.143	0.062	0.289	0.088
*pchb*	31.011	11.561	49.257	16.441
*lnays*	2.018	0.043	2.115	0.076
*lnpcgdp*	2.638	0.721	3.635	0.652
*nCohesion*	0.927	0.051	0.927	0.055
*density*	0.04	0.037	0.066	0.075
*IndStr*	0.868	0.407	0.988	0.556
*ftd*	0.152	0.298	0.353	0.574
*fiscal*	0.149	0.104	0.128	0.059
*lnHsPr*	7.596	0.562	8.098	0.561
*Adm_nCohesion*	0.869	0.071	0.856	0.079
*rdls*	0.661	0.715	0.682	0.837

Notes: Group 1 and Group 2 contain 4110 and 1254 samples, respectively.

**Table 7:** Impact of city compactness on human development: fmm iv.

	(1)	(2)
	1b. Class	2. Class
*level*	0	−0.663 ***
	(.)	(−12.21)
*region*	0	0.378 ***
	(.)	(6.89)
*_cons*	0	2.692 ***
	(.)	(10.18)
*nCohesion*	−0.304 ***	−0.359 ***
	(−9.02)	(−7.46)
*density*	−0.593 ***	−0.355 ***
	(−8.03)	(−15.21)
*IndStr*	−0.0374 ***	0.00613 **
	(−12.91)	(2.54)
*ftd*	0.0149	−0.0107 **
	(1.48)	(−3.93)
*fiscal*	−0.0252	−0.175 ***
	(−1.51)	(−11.96)
*lnHsPr*	0.0906 ***	0.146 ***
	(29.13)	(60.83)
*_cons*	−0.214 ***	−0.570 ***
	(−5.84)	(−12.05)
*var(e.hdi)*	0.00115 ***	0.00381 ***
	(35.70)	(23.50)

Notes: z statistics are in parentheses. ** and *** represents *p* < 0.05 and *p* < 0.01, respectively.

**Table 8:** Impact of city compactness on human development: robustness tests.

	(1)	(3)
	1b. Class	2. Class
*level*	0	−0.889 ***
	(.)	(−14.21)
*region*	0	−0.542 ***
	(.)	(−9.24)
*_cons*	0	3.883 ***
	(.)	(12.18)
*nCohesion*	−0.0318 **	−0.277 ***
	(−2.10)	(−9.29)
*density*	−0.313 ***	−0.489 ***
	(−11.10)	(−11.21)
*IndStr*	−0.0231 ***	−0.00570 *
	(−11.43)	(−1.75)
*ftd*	0.0106 ***	−0.0170 ***
	(2.81)	(−4.01)
*fiscal*	0.0148	−0.372 ***
	(1.56)	(−9.92)
*lnHsPr*	0.0887 ***	0.185 ***
	(49.11)	(52.45)
*rdls*	−0.0106 ***	0.0124 ***
	(−7.61)	(5.27)
*_cons*	−0.465 ***	−0.885 ***
	(−24.79)	(−25.28)
*var(e.hdi)*	0.000976 ***	0.00361 ***
	(32.07)	(22.87)

Notes: z statistics are in parentheses. *, **, and *** represents *p* < 0.1, *p* < 0.05, and *p* < 0.01, respectively.

**Table 9:** Impact of city compactness on education: fmm.

	(1)	(2)
	1b. Class	2. Class
*level*	0	−1.478 ***
	(.)	(−18.48)
*region*	0	−0.621 ***
	(.)	(−10.50)
*_cons*	0	5.237 ***
	(.)	(15.89)
*nCohesion*	−0.476 ***	−2.274 ***
	(−5.82)	(−6.94)
*density*	−1.554 ***	−1.660 ***
	(−12.45)	(−3.63)
*IndStr*	−0.0179	0.128 ***
	(−1.41)	(3.60)
*ftd*	0.0258	−0.312 ***
	(0.92)	(−8.16)
*fiscal*	−0.257 ***	−0.570 ***
	(−4.73)	(−2.87)
*lnHsPr*	0.286 ***	0.870 ***
	(28.31)	(27.79)
*_cons*	5.887 ***	3.657 ***
	(56.55)	(9.60)
*var(e.hdi)*	0.0644 ***	0.185 ***
	(31.00)	(19.01)

Notes: z statistics are in parentheses. *** represents *p* < 0.01.

**Table 10:** Impact of city compactness on health: fmm.

	(1)	(2)
	1b. Class	2. Class
*level*	0	−0.824 ***
	(.)	(−8.07)
*region*	0	−0.904 ***
	(.)	(−8.16)
*_cons*	0	2.007 ***
	(.)	(4.69)
*nCohesion*	−26.12 ***	−88.67 ***
	(−9.41)	(−3.28)
*density*	−70.50 ***	−62.04 *
	(−16.83)	(−1.72)
*IndStr*	−1.852 ***	16.17 ***
	(−5.39)	(5.72)
*ftd*	−3.161 ***	−5.927
	(−7.23)	(−1.05)
*fiscal*	6.176 ***	−26.98
	(3.75)	(−1.23)
*lnHsPr*	15.03 ***	−0.149
	(51.63)	(−0.03)
*_cons*	−53.45 ***	136.3 ***
	(−16.79)	(3.71)
*var(e.hdi)*	89.21 ***	368.0 ***
	(37.61)	(10.29)

Notes: z statistics are in parentheses. * and *** represents *p* < 0.1 and *p* < 0.01, respectively.

**Table 11:** Impact of city compactness on the standard of living: fmm.

	(1)	(2)
	1b. Class	2. Class
*level*	0	−0.625 ***
	(.)	(−7.85)
*region*	0	0.435 ***
	(.)	(4.46)
*_cons*	0	2.699 ***
	(.)	(6.08)
*nCohesion*	−0.0847	0.00338
	(−0.28)	(0.02)
*density*	−9.240 ***	−1.016 ***
	(−8.10)	(−6.30)
*IndStr*	−1.190 ***	−0.242 ***
	(−11.42)	(−14.61)
*ftd*	0.430 ***	0.104 ***
	(4.13)	(6.09)
*fiscal*	−2.095 ***	−0.714 ***
	(−6.50)	(−8.67)
*lnHsPr*	1.212 ***	1.191 ***
	(16.64)	(65.43)
*_cons*	−4.827 ***	−5.983 ***
	(−8.38)	(−36.82)
*var(e.hdi)*	0.232 ***	0.124 ***
	(14.18)	(25.27)

Notes: z statistics are in parentheses. *** represents *p* < 0.01.

## Data Availability

Data are available from the authors upon request.
